# Structure and Function Analysis of Nucleocapsid Protein of Tomato Spotted Wilt Virus Interacting with RNA Using Homology Modeling*

**DOI:** 10.1074/jbc.M114.604678

**Published:** 2014-12-24

**Authors:** Jia Li, Zhike Feng, Jianyan Wu, Ying Huang, Gang Lu, Min Zhu, Bi Wang, Xiang Mao, Xiaorong Tao

**Affiliations:** From the ‡ Department of Plant Pathology, Key Laboratory of Integrated Management of Crop Diseases and Pests (Ministry of Education), and; the ¶ College of Veterinary Medicine, Nanjing Agricultural University, Nanjing 210095 and; the § Institute of Biotechnology, Zhejiang University, Hangzhou 310029, China

**Keywords:** Homology Modeling, Negative-strand RNA Virus, Plant Virus, RNA, Virus Assembly, Tomato Spotted Wilt Virus, Nucleocapsid, Ribonucleoprotein

## Abstract

The nucleocapsid (N) protein of tomato spotted wilt virus (TSWV) plays key roles in assembling genomic RNA into ribonucleoprotein (RNP), which serves as a template for both viral gene transcription and genome replication. However, little is known about the molecular mechanism of how TSWV N interacts with genomic RNA. In this study, we demonstrated that TSWV N protein forms a range of higher ordered oligomers. Analysis of the RNA binding behavior of N protein revealed that no specific oligomer binds to RNA preferentially, instead each type of N oligomer is able to bind RNA. To better characterize the structure and function of N protein interacting with RNA, we constructed homology models of TSWV N and N-RNA complexes. Based on these homology models, we demonstrated that the positively charged and polar amino acids in its predicted surface cleft of TSWV N are critical for RNA binding. Moreover, by N-RNA homology modeling, we found that the RNA component is deeply embedded in the predicted protein cleft; consistently, TSWV N-RNA complexes are relatively resistant to digestion by RNase. Collectively, using homology modeling, we determined the RNA binding sites on N and found a new protective feature for N protein. Our findings also provide novel insights into the molecular details of the interaction of TSWV N with RNA components.

## Teaser

**Background:** The crystal structure of viral proteins is not available for many plant viruses including tomato spotted wilt virus (TSWV).

**Results:** By homology modeling, we mapped the RNA binding sites and discovered a protective feature of TSWV nucleocapsid (N).

**Conclusion:** Homology modeling provided a basis for functional analysis of TSWV N interacting with RNA.

**Significance:** This approach might be applicable for other plant viruses.

## Introduction

The *Bunyaviridae* family of negative-stranded RNA viruses comprises five genera, namely *Orthobunyavirus*, *Hantavirus*, *Phlebovirus*, *Nairovirus*, and *Tospovirus* (1). Tomato spotted wilt virus (TSWV)^2^ is the type species of the *Tospovirus* (2–4), the only genus in the family of Bunyaviridae that infects plants. TSWV causes serious diseases in numerous agronomic and ornamental crops worldwide and ranks among one of the most devastating plant viruses (5). Like other members of the Bunyaviridae, the TSWV viral particle is membrane enveloped and spherical. The genome of TSWV consists of three negative-sense single-stranded RNAs (ssRNAs) named L, M, and S. The genomic L RNA encodes RNA-dependent RNA polymerase (RdRp) using a negative coding strategy (6, 7). The genomic M and S RNAs encode open reading frames (ORFs) using ambisense coding strategy. The genomic M encodes glycoproteins Gn and Gc from the viral complementary RNA and encodes the movement protein (NSm) from the viral RNA (8). The genomic S encodes nucleoprotein (N) from the viral complementary RNA and nonstructural protein (NSs) from the viral complementary RNA, which functions as a viral suppressor of RNA silencing (9, 10).

Nucleocapsid (N) of TSWV is the main structural protein that assembles the genomic RNAs into ribonucleoprotein complexes (RNPs) (11). The RNPs are central to the viral cycle of TSWV and other Bunyaviruses because only the RNPs, but not the naked RNAs, serve as the template for viral genome replication and gene transcription (3). Using yeast two-hybrid assay, TSWV N protein monomers was shown to interact with each other in a head-to-tail fashion (12). The TSWV N protein was also shown to bind single-stranded RNA irrespective of sequence (13). However, the molecular details of TSWV N-N multimerization and the interaction of TSWV N oligomers with genomic RNA remain to be elucidated. Although both halves of N were shown to be involved in RNA binding (13), the RNA binding sites on the N protein also remain to be determined. The TSWV genomic RNA is always associated with N protein; however, little is known about the protective function of the N-RNA complexes.

Tremendous progress in determining the crystal structure of viral proteins from human and animal bunyaviruses has been made in recent years. The N protein structures from Rift Valley fever virus (genus *Phlebovirus*) (14–16), Crimean-Congo hemorrhagic fever virus (genus *Nairovirus*) (17, 18), and four members in the genus *Orthobunyavirus*: bunyawera virus (19), Schmallenberg virus (20), Leanyer virus (21), and La Crosse virus (22), have all been determined. When the crystal structure is known, then the biological function of the viral protein can be readily elucidated. However, determination of the protein structures of plant viruses, including plant negative-stranded RNA viruses, was progressed far less than for mammalian viruses. There are more than 20 species of viruses in the *Tospovirus* genus (11), but none of the protein structures for these viruses have been determined. The sequence identity or similarity of N protein between TSWV and mammalian bunyaviruses is quite low (8–12% amino acid sequence similarity). Without protein structures, functional analysis has proven to be very difficult. Fortunately, there are many homology modeling approaches currently available using amino acid sequence similarity or multiple threading alignments (23–26). With a homology modeled structure, we would have a molecular basis to design experiments to evaluate the structure and analyze the function of the viral protein.

In this study, we developed blue native gel analysis, homology modeling, and other approaches to investigate the TSWV N-N oligomerization and the interaction of TSWV N protein with genomic RNA. We demonstrated that the TSWV N protein forms a range of higher ordered oligomers. Analysis of the RNA binding behavior of N protein using two different approaches revealed that no specific oligomer binds to RNA preferentially, instead each type of N oligomer is able to associate with RNA. To better characterize the N protein in its interaction with genomic RNA, we constructed homology models of TSWV N and N-RNA complexes. Based on these homology models, we mapped the RNA binding sites onto its predicted surface cleft of TSWV N. Moreover, by N-RNA homology modeling we found that the RNA component is deeply embedded in the protein cleft of N; consistently, RNase A treatment assay revealed that TSWV N-RNA complexes are relatively resistant to digestion by RNase. Taken together, using homology modeling we determined the RNA binding sites on the TSWV N protein and obtained new insights into the protection function of N-RNA complexes.

## EXPERIMENTAL PROCEDURES

### 

#### 

##### Construction of Plasmids

The coding region of the N protein was amplified from the cDNA of TSWV Yunnan isolate (accession JF960235.1) (27) using forward primer 5′-CC**CATATG**TCTAAGGTTAAGCTCAC-3′ and reverse primer 5′-GG**GAGCTC**TTAAGCAAGTTCTGCAAGT-3′ (nucleotide sequences in bold represent the NdeI and SacI restriction site, respectively). The DNA amplicon was digested with NdeI and SacI restriction enzymes and cloned into modified bacterial expression vector pET28a (modified to contain with a tobacco etch virus cleavage site) downstream of the His_6_ tag. The alanine substitution mutations of N protein on Arg^60^, Lys^65^/Lys^68^, Lys^81^, Arg^94^/Arg^95^, Lys^183^/Tyr^184^, Lys^192^/Thr^195^ were created by PCR-based site-directed mutagenesis based on pET28-N for bacterial expression. The entire coding region of each mutant was confirmed by DNA sequencing. The RNA transcription plasmid pMD19-T7-5′UTR-GFP-3′UTR with the TSWV termini sequence was constructed by cloning the coding region of green fluorescent protein (GFP) between the TSWV S segment 5′- and 3′-untranslated region (UTR) under the control of the T7 promoter, then cloning it into the pMD19 vector (TakaRa, Dalian, China).

##### Protein Expression and Purification

*Escherichia coli* Rosetta (DE3) was transformed with pET28-N and its derivatives. For expressing proteins, a small overnight culture (10 ml) was transferred to a 1-liter culture and allowed to grow at 37 °C until the optical density at 600 nm reached 0.6. Protein expression was then induced with 0.1 mm isopropyl β-d-thiogalactopyranoside overnight at 25 °C. The cells were harvested and resuspended in cold lysis buffer (50 mm NaH_2_PO_4_, 300 mm NaCl, 10 mm imidazole, pH 8.0). After pre-treatment with lysozyme for 30 min, cells were lysed by sonication for 20 min on ice. Cell debris was removed by centrifugation at 12,800 × *g* for 1 h. The supernatant was incubated with 1 ml of nickel-nitrilotriacetic acid resin (Ni-NTA, Qiagen, Hilden, Germany) at 4 °C for 2 h and then loaded onto a chromatographic column (Bio-Rad). The Ni-NTA resin was washed three times with 50 mm wash buffer (50 mm NaH_2_PO_4_, 300 mm NaCl, 50 mm imidazole, pH 8.0). The proteins were eluted with 250 mm imidazole elution buffer (50 mm NaH_2_PO_4_, 300 mm NaCl, 250 mm imidazole, pH 8.0). The protein was dialyzed in storage buffer (50 mm NaH_2_PO_4_, 300 mm NaCl, pH 8.0) and stored at −70 °C until use. To purify the free-tag TSWV N protein, we removed the N-terminal His_6_ tag from the recombinant N protein, which was expressed from pET28a-N by a recombinant His_6_-tobacco etch virus protease as described by Wu *et al.* (28).

To remove the endogenous *E. coli* RNA in recombinant N protein, the *E. coli* lysate was treated with polyethyleneimine (PEI, Sigma). PEI was added slowly into the lysate with stirring until the final concentration reached 0.1% (w/v). The nucleic acids were then removed by centrifugation at 12,800 × *g* for 15 min. The supernatant was further purified by Ni-NTA affinity chromatography.

##### RNA Extraction and Analysis

Total RNA was isolated from the N-RNA complex of the wild type and mutant proteins by adding 2× RNA gel loading buffer containing 95% formamide, which effectively denatures and dissociates RNA from the protein. After boiling at 100 °C for 5 min, the samples were electrophoresed in 1.0% nondenaturing agarose. RNAs were stained with ethidium bromide and visualized using long wavelength UV light. For analysis of the cellular RNA bound to each oligomeric type of N protein, the proteins were recovered from the blue native gel and mixed with RNA loading buffer to release the RNA and analyzed in 1.0% agarose gel.

##### Discontinuous Blue Native Gel Electrophoresis Analysis

The blue native gel electrophoresis analysis was carried out as described by Wu *et al.* (28). Briefly, purified proteins were mixed with BN-PAGE sample buffer (100 mm Tris-Cl, pH 8.0, 40% glycerol, 0.5% Coomassie Brilliant Blue G-250) and incubated for 10 min at room temperature. Protein samples were loaded onto 4–16% discontinuous blue native PAGE gels and separated at 4 °C in a cathode buffer containing 100 mm histidine (adjusted to pH 8.0 using Tris base) and 0.002% Coomassie Brilliant Blue G-250. The gels were fixed and destained in 7.5% acetic acid and 5% ethanol. For isolation of different oligomers from blue native gels, the corresponding band was precisely cut from the native gels immediately after electrophoresis was completed, the gel was minced, and the protein was eluted in phosphate-buffered saline (PBS). For analyzing the oligomeric dynamics of N protein, each recovered oligomeric type of N protein was run in a new 4–16% discontinuous native PAGE gels.

##### Cross-linking

The purified N protein (30 μg) was mixed with various concentrations of freshly prepared glutaraldehyde solution, then incubated at 37 °C for 5 min. The reactions were quenched by adding 2 μl of 1 m Tris-HCl, pH 7.5. The protein samples were then boiled in SDS-denaturing buffer, and separated in 5–20% SDS-polyacrylamide gradient gels. Proteins were detected by Coomassie Blue staining.

##### Preparation of RNA Transcripts by in Vitro Transcription

RNA transcripts were generated using the T7 Riboprobe *in vitro* Transcription kit (Promega, Madison, WI). RNA transcripts were synthesized in a reaction mixture containing 2.5 mm (each) ATP, CTP, GTP, and UTP. DIG-labeled riboprobes were synthesized in 20-μl reaction volumes containing 1 mm NTP or 1 mm (each) ATP, CTP, and GTP, 0.65 mm UTP, 0.35 mm DIG-11-UTP (Roche Applied Science), 500 ng of linearized template, 20 units of RNase inhibitor, and 40 units of T7 polymerase and incubated at 37 °C for 2 to 3 h. All reaction mixtures were then treated with 1 μl of DNase I for 15 min at 37 °C. The transcripts were purified by phenol-chloroform extraction and ethanol precipitation in the presence of ammonium acetate and then dissolved in RNase-free, double-distilled H_2_O (ddH_2_O). The concentrations of RNA transcripts were determined by measuring absorption at *A*_260_ with a NanoDrop 1000 spectrophotometer (Thermo Electron, MA).

##### Western Blot Analysis

Protein concentrations were determined using the Bio-Rad protein assay kit. Protein samples were treated with 3× SDS buffer. After boiling for 7 min, the samples were separated by electrophoresis in 10% SDS-polyacrylamide gels and transferred onto a nitrocellulose membrane. The antigens on the membrane were incubated with a monoclonal antibody against TSWV N (1:10,000 dilution) (29) and subsequently probed with AP-coupled goat anti-mouse IgG (1:10,000 dilution; Sigma). The signals on the membrane were visualized with ready-for-use 5-bromo-4-chloro-3-indolylphosphate/nitroblue tetrazolium (BCIP/NBT) solution (Sangon Biotech, Shanghai, China).

##### Construction of Homology Model of TSWV N and N-RNA Complexes

The homology model of TSWV N protein was constructed using the on-line server at I-TASSER (25, 26). The N structure bound with RNA was constructed by modeling an 11-nucleotide (nt) RNA from the crystal structure of Bunyawera virus N protein onto the homology model of N. Visualization and display of the amino acid residues and electrostatic surface potential on the homology structure were carried out using the PyMOL Molecular Graphics System, version 1.5.0.4, Schrödinger, LLC.

##### Filter Binding Assays

The dissociation constants for TSWV N and mutants were determined by filter binding assay. The DIG-labeled TSWV RNA probe (5′UTR-GFP-3′UTR, 3 nm) was mixed with increasing concentrations of wild type or mutant N proteins. Reaction mixtures were incubated at room temperature for 10 min and filtered through two layers of membranes under vacuum: the top membrane (nitrocellulose) binds proteins and RNA-protein complexes, and the bottom membrane (nylon which is charged) collects free RNA. Filters were washed with 10 ml of RNA binding buffer. The amount of RNA retained on the filter was detected by digoxigenin-AP Fab fragments (Roche Applied Science) followed by BCIP/NBT visualization. The bound RNA was quantified as described by Wu *et al.* (28).

##### Electrophoretic Mobility Shift Assay

In a typical EMSA, increasing amounts of N or mutants proteins were incubated in 10 μl of binding buffer (45 mm Tris, 45 mm boric acid, pH 8.4) with 3 nmol of DIG-11-UTP-labeled RNA probe (5′UTR-GFP-3′UTR). The reaction mixtures were incubated for 10 min at room temperature, then 5 μl of loading buffer (0.1% bromphenol blue, 0.1% xylene cyanole, 15% Ficoll 400 in 0.5× TBE buffer) was added. The reaction mixtures were separated by electrophoresis in 1% agarose with 0.5× TBE (45 mm Tris, 45 mm boric acid, 1 mm EDTA, pH 8.0). The gel was electroblotted onto a nylon membrane (Hybond N^+^; GE Healthcare). The electrophoretic retardation of the DIG-labeled RNAs was detected by AP-labeled anti-digoxigenin followed with BCIP/NBT staining.

##### RNase A Treatment Assay

The DIG-labeled RNA probe with the TSWV termini (5′UTR-GFP-3′UTR, 3 nmol) was incubated in the presence or absence of 2 μg of TSWV N or non-RNA binding mutant protein in a total reaction volume of 10 μl in binding buffer (45 mm Tris, 45 mm boric acid, pH 8.4). The reaction mixtures were incubated at room temperature for 10 min. RNase A was then added to the concentrations specified, and the mixtures were further incubated at 37 °C for 30 min. The products of the binding reactions were then separated by electrophoresis in 1% agarose. The gel was electroblotted onto a nylon membrane (Hybond N^+^; GE Healthcare). The RNA on the membranes was fixed by 0.25 J/cm^2^ of UV light (UVP, CA). The effects of RNase A on the N-RNA complexes or mutant-RNA complexes on the membrane was detected using AP-linked anti-digoxigenin antibody, followed by signal development in ready-for-use BCIP/NBT solution.

## RESULTS

### 

#### 

##### Expression and Purification of Recombinant TSWV N Protein

**FIGURE 1.**
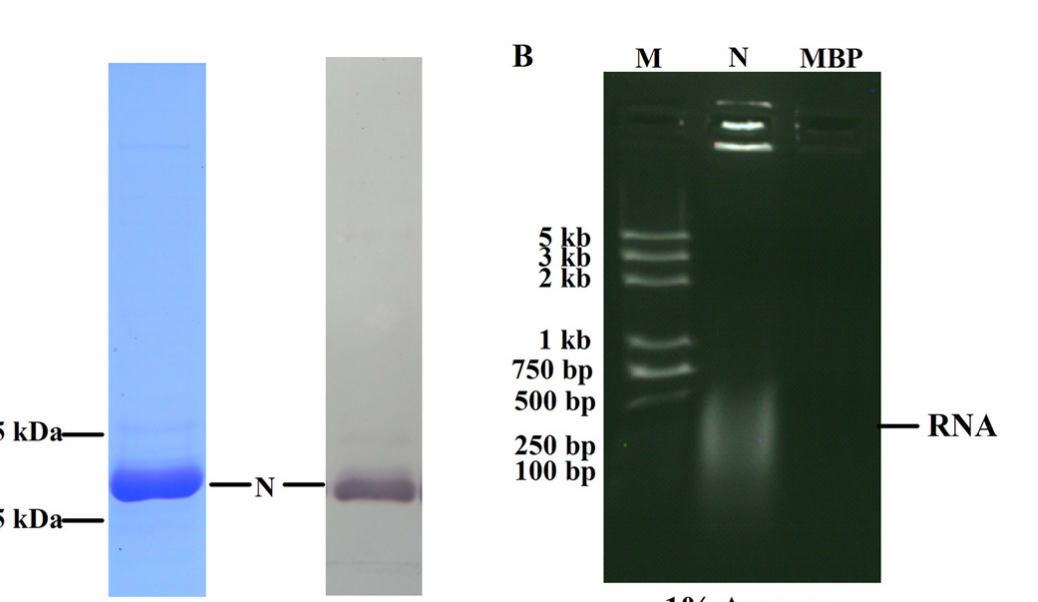
**Recombinant TSWV N protein binds *E. coli* cellular RNA.** *A,* expression and purification of the recombinant N protein. Purified N protein was analyzed by 10% SDS-PAGE stained with Coomassie Blue (*left*, 10 μg of N protein) or detected by Western blot (*right*, 100 ng of N protein) using a monoclonal antibody (1:10,000 dilution) raised against the N protein. *B,* gel electrophoresis analysis of cellular RNA extracted from the purified N protein. The cellular RNAs were denatured and released from purified N or recombinant maltose-binding protein (*MBP*) and electrophoresed on a 1% agarose gel using ethidium bromide staining. RNA is marked with an *arrow*.

To purify the TSWV N protein for biochemical characterization, we fused the N protein with the N-terminal His_6_ tag for expression in *E. coli*. After isopropyl β-d-thiogalactopyranoside induction, the recombinant N protein was purified under native conditions by nickel affinity chromatography. Analysis of the recombinant protein by SDS-PAGE followed with Coomassie Blue staining showed a specific band at the size expected for the His_6_-tagged N protein (31 kDa). Further analysis by Western blot using the monoclonal antibody raised against the TSWV N protein confirmed the identity of the recombinant protein as TSWV N (Fig. 1*A*).

When we roughly determined the concentration of purified protein using a UV spectrophotometer, we found that the ratio of the *A* at 260 to 280 nm (*A*_260_/*A*_280_) for the purified TSWV N protein was up to 1.7 (data not shown), which indicated that the recombinant TSWV N protein was bound to *E. coli* endogenous RNA. To substantiate this observation, we denatured the purified N protein and analyzed the released RNA in a 1% agarose gel. We included a recombinant maltose-binding protein expressed in the same strain of *E. coli* as a control. As shown in Fig. 1*B*, the N protein was readily detected with RNA in the agarose gel staining with ethidium bromide, whereas the recombinant maltose-binding protein had no detected cellular RNA. These results suggest that the TSWV N protein binds nonspecifically to *E. coli* cellular RNA. The observation of nonspecific binding of TSWV N protein to RNA is consistent with a previous report that N protein binds to RNA irrespective of sequence as described by Richmond *et al.* (13).

##### Oligomerization State of N Protein

**FIGURE 2.**
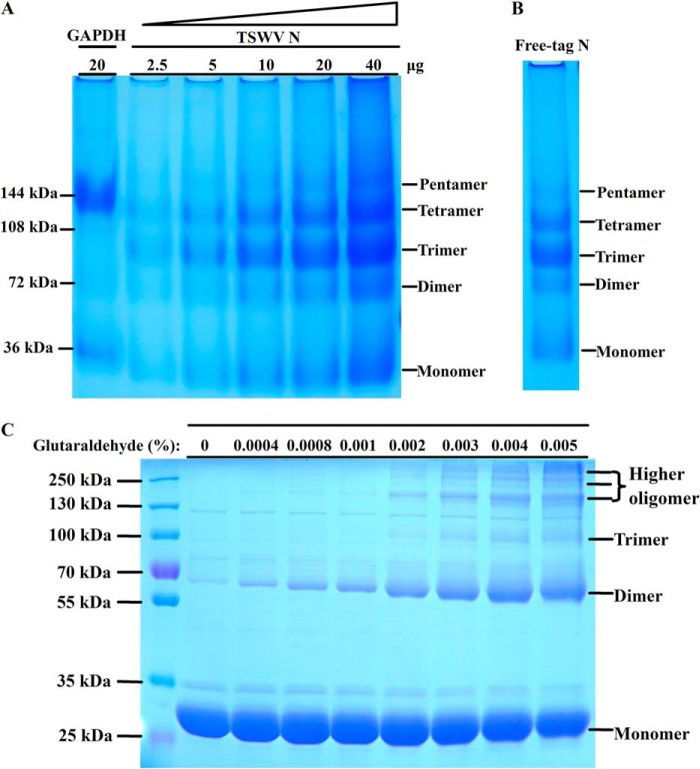
**Oligomerization state analysis of TSWV N protein.** *A*, TSWV N protein forms a range of higher ordered oligomers in discontinuous blue native gel. Increasing amounts (2.5, 5, 10, 20, and 40 μg) of purified TSWV N were separated on a 4–16% discontinuous polyacrylamide gel. GAPDH (20 μg) was used as a size marker. *B,* discontinuous blue native PAGE analysis of oligomerization state of free-tag N. Approximately 20 μg of free-tag N was separated on a 4–16% discontinuous polyacrylamide gel. *C,* cross-linking of N protein by glutaraldehyde. The 30 μg of purified N protein was incubated with increasing amounts of glutaraldehyde. The mixtures were incubated at 37 °C for 5 min and then separated by 5–20% SDS-PAGE followed by Coomassie Blue staining. The protein marker was loaded on the same gel to mark the size of cross-linking products. Monomer, dimer, trimer, tetramer, and higher ordered oligomer positions are indicated in each gel.

To investigate the oligomerization state of TSWV N, we used a discontinuous blue native gel electrophoresis analysis, which allows the separation of the protein complex in its native quaternary structure according to its size and shape (28, 30–33). We analyzed the purified N protein with 4–16% discontinuous blue native PAGE. As shown in Fig. 2*A*, TSWV N forms a range of oligomeric bands with a molecular size of 31, 62, 93, 124, and 155 kDa, which correspond to the monomer, dimer, trimer, tetramer, and pentamer of the N protein, respectively. The N proteins used in the above assay were purified by Ni-NTA resin and contained an extra His_6_ tag on its N terminus. To ascertain whether the extra His_6_ tag at its N terminus affects the oligomerization state of N, we also generated free-tag N protein. As shown in blue native PAGE, the free-tag N protein formed a pattern of monomers, dimers, trimers, and tetramers similar to those of His_6_-tagged N protein (Fig. 2, *A* and *B*). To further examine the oligomerization state of the N protein, we cross-linked the recombinant N protein by adding increasing amounts of glutaraldehyde. As the concentration of glutaraldehyde increased, an intensified band of dimers, trimers, and higher oligomers appeared (Fig. 2*C*), confirming that the N protein was able to form different higher ordered oligomers. Hence, TSWV N was able to form a range of higher ordered oligomers.

##### RNA Binding Behavior of N Protein Oligomers

**FIGURE 3.**
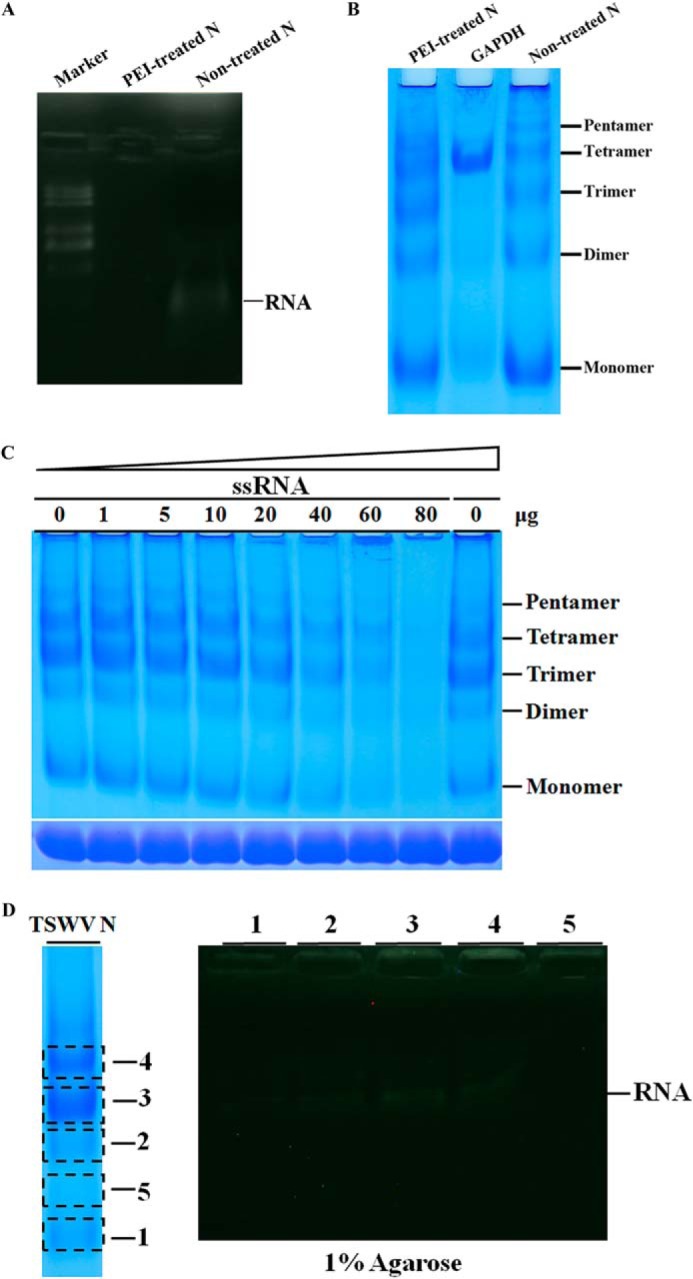
**RNA binding behavior of TSWV N protein.** *A,* gel electrophoresis analysis of cellular RNA extracted from PEI-treated N protein. The cellular RNAs were denatured and released from PEI-treated or non-treated N protein and electrophoresed on a 1% agarose gel using ethidium bromide staining. RNA is marked with an *arrow. B,* discontinuous blue native PAGE analysis of oligomeric formation of PEI-treated N protein. PEI-treated N protein was loaded together with a non-treated N protein in a 4–16% discontinuous blue native PAGE. GAPDH (20 μg) was used as a size marker. The positions of the monomer, dimer, trimer, tetramer, and pentamer are indicated. *C,* analysis of RNA binding behavior of N protein by gel mobility shift assay in a discontinuous blue native PAGE. A fixed amount of N (15 μg) was incubated with increasing amounts of unlabeled RNA transcripts with viral termini (5′UTR-GFP-3′UTR), and the complexes were resolved in a 4–16% polyacrylamide gradient gel (*upper gel*). The same samples were also analyzed in a 10% SDS-PAGE gel (*lower gel*). *D,* analysis of cellular RNA bound in different types of N oligomers. Purified N protein was separated in 4–16% discontinuous blue native PAGE (*left panel*). The bands of monomers, dimers, trimers, and tetramers (corresponding to numbers 1–4, respectively) or a region of the gel that does not contain N proteins (*number 5*) were precisely excised, minced, and protein in the gel was eluted with PBS. The recovered samples were denatured in RNA loading buffer containing formamide and boiled for 5 min. The cellular RNA released from N-RNA complex was analyzed in a 1% agarose by ethidium bromide staining (*right panel*). RNA is marked with an *arrow*.

Given that N protein formed different types of higher ordered oligomers, we next determined the RNA binding behavior of N protein oligomers. Because N protein binds to *E. coli* RNA nonspecifically, we first removed the *E. coli* RNA from the N protein by PEI treatment. RNA gel analysis showed that PEI-treated N protein was largely RNA-free (Fig. 3*A*). To check the oligomeric formation of RNA-free N protein, we determined the PEI-treated protein along with N protein without PEI treatment in the discontinuous blue native gel. PEI-treated N protein formed a similar pattern of higher ordered oligomers compared with those of N protein without treatment (Fig. 3*B*). It strongly suggests that higher ordered oligomeric formation of the N protein does not depend on the binding of RNA. The bands of higher ordered oligomers formed by PEI-treated protein ran faster than those by the non-treated protein, which may be due to the smaller size of complexes formed by RNA-free N protein. We next carried out an electrophoresis mobility shift assay to analyze the binding behavior of N oligomers in the discontinuous blue native gel. A fixed amount of PEI-treated N protein (15 μg) was incubated with increasing amounts of RNA transcripts with the TSWV termini sequence representing the 5′-UTR and 3′-UTR of the TSWV genomic S fused with GFP at both ends (5′UTR-GFP-3′UTR, 961 nt), and the reaction mixtures were analyzed in a 4–16% blue native polyacrylamide gradient gel. As shown in Fig. 3*C* (*upper gel*), when 1 to 10 μg of RNA was added, the oligomers of N did not shift in an obvious manner. At 20 to 80 μg of RNA, the monomer, dimer, trimer, tetramer, and pentamer bands all disappear. The disappearance of those bands strongly implies that they bind RNA to form higher ordered protein-RNA complexes. To rule out the possibility that the disappearance of protein bands is due to the contamination of protease in the synthesized RNAs, the same protein-RNA complexes were also analyzed in 10% denatured SDS-PAGE gel. No degradation was found for those protein complexes (Fig. 3*C*, *lower gel*).

The above experiments suggest that each type of TSWV N oligomer may be able to bind RNA. To substantiate this observation, we took advantage of the features that TSWV N protein binds to *E. coli* cellular RNA. The purified N protein was separated by 4–16% discontinuous blue native PAGE. Each type of N oligomers (number 1–4) and a region of the gel that does not contain N protein (number 5) was isolated from the native gel, respectively (Fig. 3*D*, *left panel*). The recovered protein was denatured to release the cellular RNA, then separated in a 1% agarose gel. As shown in Fig. 3*D* (*right panel*), the monomers, dimers, trimers, and tetramers all contained *E. coli* cellular RNA, whereas the region of the gel without N protein did not contain RNA, suggesting that each type of N oligomer was able to bind RNA.

##### Construction of the Homology Model of TSWV N and N-RNA Complex

An earlier study reported that both the N amino and carboxyl halves of TSWV N are involved in binding to RNA (13). However, precisely determining the RNA binding sites of N protein turned out to be very challenging because the positively charged amino acids that may be responsible for the RNA binding were so abundant and distributed throughout the entire N polypeptide.

**FIGURE 4.**
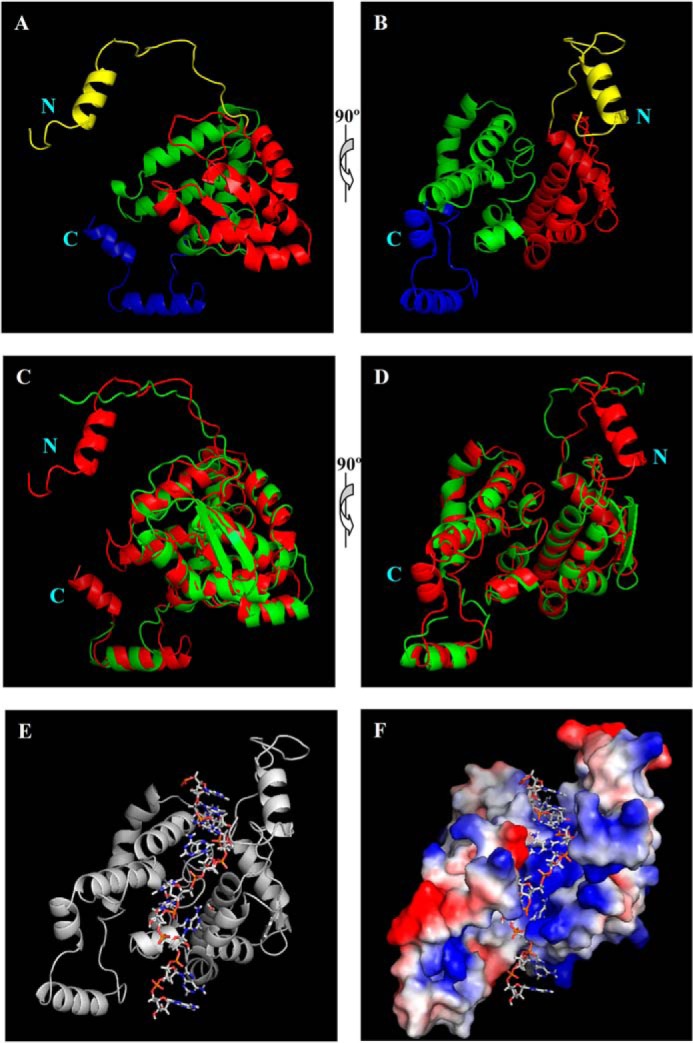
**Homology models of TSWV N and N-RNA complexes.** *A* and *B,* the three-dimensional structure of the TSWV N protein by homology modeling. The homology model of TSWV N was constructed by I-TASSER. Predicted three-dimensional structure of TSWV N consists of the N-terminal arm (*yellow*), N-terminal domain (*red*), C-terminal domain (*green*), and C-terminal arm (*blue*). The diagrams in *panel B* were generated after the image in *A* was rotated 90°. *C* and *D,* superposition of homology structure of TSWV N (*red*) on the crystal structure of Bunyamwera virus N (*green*). Image *D* was generated by rotating the image in *C* by 90°. *E,* the three-dimensional structure of TSWV N-RNA complexes. An 11-nt RNA was modeled onto the homology model of the TSWV N protein. TSWV N protomer is shown as a *ribbon*, and RNA is shown as *sticks. F,* electrostatic surface potential of the homology model of N protein bound to RNA (*sticks*). Positively charged areas are in *blue*; negatively charged areas are in *red*.

To better characterize the RNA binding sites and other features of the N protein in its interaction with RNA, we built a three-dimensional homology model of TSWV N protein using an on-line platform for protein structure and function predictions, I-TASSER, based on multiple threading alignments. As shown in Fig. 4, *A* and *B*, the homology structure of N protein has four parts: the N-arm (*yellow*), N-terminal domain (*red*), C-terminal domain (*green*), and C-arm (*blue*). The N-/C-arms comprised sequences from the N and C termini, which may play an important role in mediating the N-N interaction. The N- and C-terminal domains of the N protein forms a core structure. Displaying the surface of the N- and C-terminal domains demonstrated that a protein cleft was formed between the N- and C-terminal domains (Fig. 4*E*). Superposition of the homology structure of TSWV N onto the crystal structure of Bunyawera virus N (19) showed a remarkable similarity of overall structure-folding between the two structures. The main differences are in the positions of the N- and C-arms (Fig. 4, *C* and *D*).

Analysis of the electrostatic surface potential of N homology structure showed a charged surface cleft (*blue* in Fig. 4*F*), which may be responsible for RNA binding. We then built an 11-nt RNA into the homology model of the N protein. In the homology model of the N-RNA complexes, the RNA fit well into the surface cleft of the N protein. The RNA component is deeply embedded in the protein cleft (Fig. 4, *E* and *F*), suggesting a possible structure protection for the packaged RNA.

##### Mapping the RNA Binding Sites on TSWV N Protein

**FIGURE 5.**
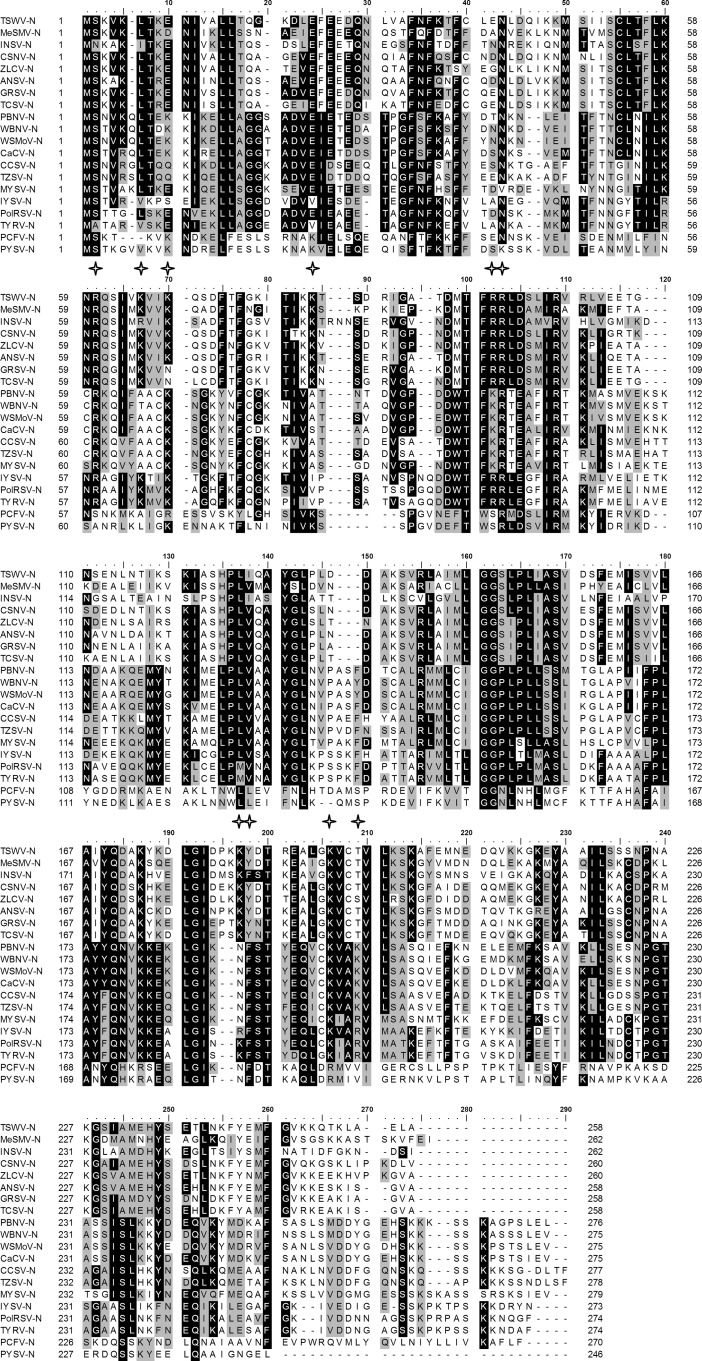
**Alignment of N protein sequences reveals conserved amino acids within Tospoviruses.** ClustalX was used for the multiple alignment of the N protein sequence from 20 nucleocapsid protein sequences from GenBank: TSWV (JF960235.1); melon severe mosaic virus (*MeSMV*, EU275149), impatiens necrotic spot virus (*INSV*, NC_003624), chrysanthemum stem necrosis virus (*CSNV*, AF067068), zucchini lethal chlorosis virus (*ZLCV*, AF067069), alstroemeria necrotic streak virus (*ANSV*, GQ478668), groundnut ringspot virus (*GRSV*, L12048), tomato chlorotic spot virus (*TCSV*, S54325), peanut bud necrosis virus (*PBNV*, U27809), watermelon bud necrosis virus (*WBNV*, GU584184), watermelon silver mottle virus (*WSMoV*, U78734), capsicum chlorosis virus (*CaCV*, NC_008301), calla lily chlorotic spot virus (*CCSV*, AY867502), tomato zonate spot virus (*TZSV*, NC_010489), melon yellow spot virus (*MYSV*, AB038343), iris yellow spot virus (*IYSV*, AF001387), polygonum ringspot virus (*PolRSV*, EF445397), tomato yellow ring virus (*TYRV*, AY686718), peanut chlorotic fan-spot virus (*PCFV*, AF080526), and peanut yellow spot virus (*PYSV*, AF013994). The N protein can be divided into two groups: Euro-Asia and America groups. Positions of Arg^60^, Lys^65^/Lys^68^, Lys^81^, Arg^94^/Arg^95^, Lys^183^/Tyr^184^, and Lys^192^/Thr^195^ on TSWV N selected for alanine substitutions are indicated.

**FIGURE 6.**
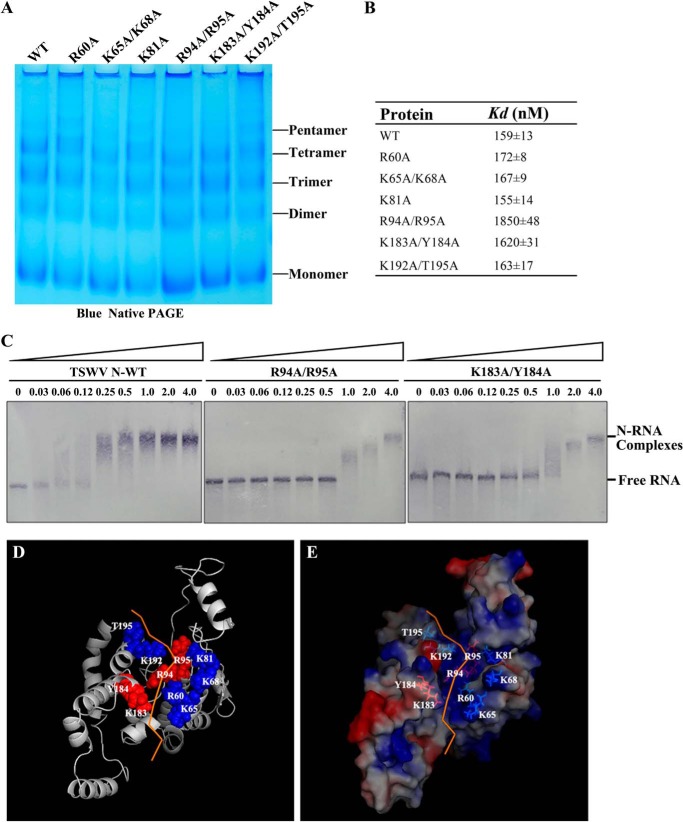
**Positively charged and polar amino acid residues in the predicted surface cleft of the N are important for RNA binding.** *A,* oligomeric formation analysis of alanine-substituted proteins using a discontinuous blue native PAGE. Approximately 15 μg of each PEI-treated mutant protein was loaded on a 4–16% discontinuous polyacrylamide gel. The same amount of wild type N protein was loaded as a control. Monomers, dimers, trimers, and tetramers are indicated. *B,* dissociation constants for the association of wild type and mutant N proteins. Increasing concentrations of N or mutant protein were incubated with DIG-labeled 5′UTR-GFP-3′UTR RNA transcripts. RNA-protein complexes were assayed and quantified by nitrocellulose filter binding with DIG-labeled RNA followed by AP-linked anti-DIG antibody and BCIP/NBT visualization. *C,* RNA binding analysis of R94A/R95A and K183A/Y184A mutants by gel mobility shift assay. DIG-labeled RNA (3 nmol) was incubated with increasing amounts of N mutants, and mixtures were applied to a 1% agarose gel. The blots were detected by anti-DIG AP-linked monoclonal antibody. *D* and *E,* displaying the RNA binding sites on the three-dimensional structure of homology model of N protein. The three-dimensional structure of the N homology model is shown as a schematic (*D*) and surface representation (*E*). The RNA is displayed as an *orange ribbon*. Amino acid residues Arg^94^, Arg^95^, Lys^183^, and Tyr^184^ are each labeled with *red spheres*; residues Arg^60^, Lys^65^, Lys^68^, Lys^81^, Lys^192^, and Thr^195^ are labeled with *blue spheres*.

The homology model of the TSWV N structure suggested that the charged surface cleft of the N protein may be responsible for RNA binding. To determine the amino acids of N protein that are involved in RNA binding, we obtained nucleocapsid sequences from the 20 *Tospoviruses* in GenBank^TM^ and aligned the sequences. The positive-charged and polar amino acids, which are conserved in the N protein sequence (Fig. 5) and located in the protein cleft of the N homology model (Fig. 4, *E* and *F*), were selected for site-directed alanine substitution mutagenesis. We then generated protein mutants R60A, K65A/K68A, K81A, R94A/R95A, K183A/Y184A, and K192A/T195A, respectively. All N alanine substitution mutants were expressed in *E. coli*, treated with PEI, and purified using Ni-NTA affinity chromatography. The oligomeric formation of alanine-substitution mutant proteins was examined in the 4–16% discontinuous blue native gel. As shown in Fig. 6*A*, all N alanine-substitution mutants displayed a similar pattern of oligomeric formation compared with the wild-type N protein, suggesting that oligomeric formation by the mutants did not change substantially.

To examine whether any mutant was defective in RNA binding or reduced in their binding ability, we determined the dissociation constant for each of alanine-substitution mutant using a filter binding assay. A fixed amount of DIG-labeled RNA containing the TSWV termini sequence (5′UTR-GFP-3′UTR, 3 nmol) was mixed with increasing quantities of wild-type N or mutant proteins. Quantification analysis of the RNA-protein complexes retained on nitrocellulose showed that the dissociation constant for mutants R60A, K65A/K68A, K81A, and K192A/T195A was comparable with the wild-type N protein; however, the dissociation constant for mutants R94A/R95A and K183A/Y184A was greater than that of wild-type N protein (Fig. 6*B*), suggesting that the RNA binding affinity of both R94A/R95A and K183A/Y184A was significantly reduced. To verify this observation, we further performed a gel mobility assay for these two mutants. A fixed amount of DIG-labeled RNA transcripts (3 nmol) was incubated with increasing concentrations of R94A/R95A and K183A/Y184A mutant protein, respectively. Compared with the wild-type protein, R94A/R95A and K183A/Y184A had significantly less RNA-binding ability in a gel mobility assay (Fig. 6*C*). These data demonstrated that amino acids Arg^94^, Arg^95^, Lys^183^, and Tyr^184^ are important for N binding to RNA. When those amino acids are displayed in the three-dimensional structure of N homology model, they mapped onto the surface cleft of N protein (Fig. 6, *D* and *E*).

##### RNase Resistant Assay for RNA Complexed with TSWV N Protein

**FIGURE 7.**
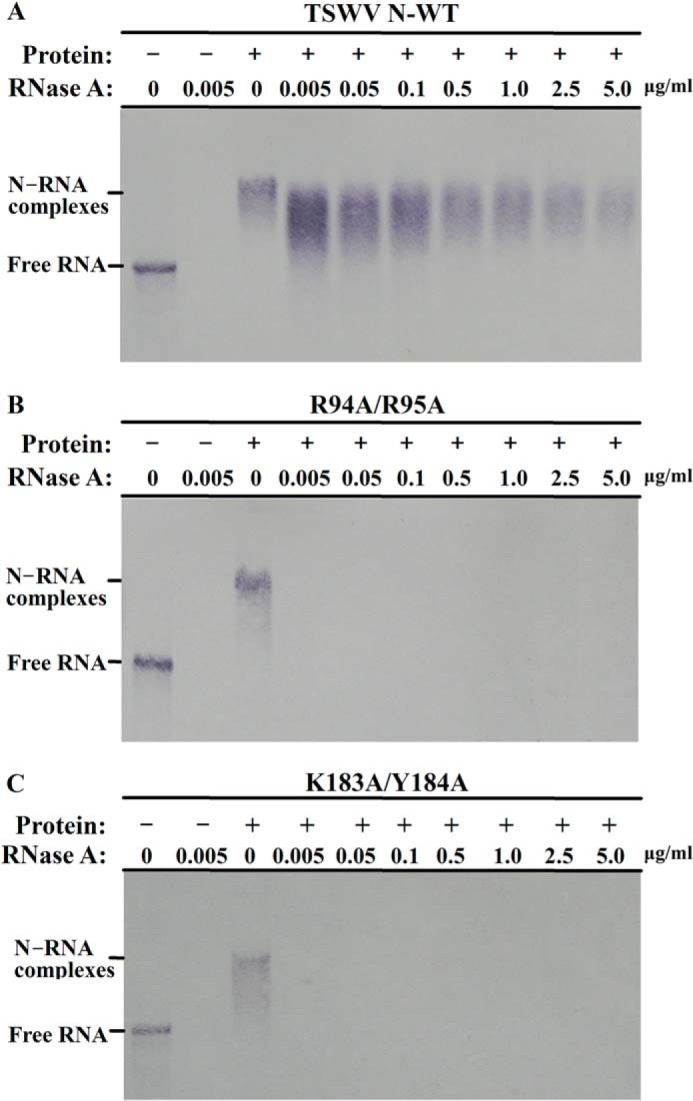
**RNA complexed with TSWV N protein is partially resistant to RNase.** RNase A treatment assay for RNA complexed with wild type N (*A*), R94A/R95A (*B*), or K183A/Y184A mutant (*C*). The DIG-labeled riboprobe representing RNA with viral termini (10 ng) was incubated with wild type or mutant N proteins (2 μg) for 10 min at room temperature to form an RNA-N complex. Then increasing amounts of RNase A were added, and the digestion reaction ran for 30 min at 37 °C. The DIG-labeled probe (10 ng) alone was also treated with or without 0.005 μg/ml of RNase A. The samples were resolved in a 1% agarose gel, and the DIG-labeled RNA on the blots was visualized with AP-linked anti-DIG antibody and BCIP/NBT.

From the homology model of N-RNA complexes, we observed that the modeled 11-nt RNA component is deeply embedded in the protein cleft. Such a structure may protect the RNA from digestion by RNase. To address whether the TSWV N was indeed able to protect the RNA by the degradation of RNase, DIG-labeled riboprobe (5′UTR-GFP-3′UTR, 10 ng) was incubated with 2 μg of PEI-treated N protein to form N-RNA complexes, then increasing amounts of RNase A were added. When 0.005 μg/ml of RNase A was added, the band of N-RNA complexes became broader and shifted a little lower, which may be due to partial digestion by RNA in N-RNA complexes. However, the remaining RNA complexed with the TSWV N protein was still resistant to RNase A. When the concentration of RNase A was increasing, RNA complexed with the N protein was partially resistant to digestion with up to 5 μg/ml of RNase A (Fig. 7*A*). To ensure that the N protein-RNA interaction, which is directly providing resistance to RNase A, non-RNA binding variants of the N protein, R94A/R95A, and K183A/Y184A, were used for the RNA protection assay, respectively. DIG-labeled riboprobe (5′UTR-GFP-3′UTR, 10 ng) was incubated with 2 μg of R94A/R95A or K183A/Y184A mutant protein, then increasing amounts of RNase A were added. As shown in Fig. 7, *B* and *C*, the addition of 0.005 μg/ml of RNase A completely digested the RNA from mutant-RNA complexes, implying that the mutant that lost the RNA-binding activity was no longer able to protect the RNA from RNase treatment. In summary, these results suggested that the TSWV N protein can protect RNA from degradation by RNase.

## DISCUSSION

In this paper, we reported structure and function analysis of TSWV N interacting with RNA using homology modeling. Based on homology models of TSWV N and N-RNA complexes, we mapped RNA binding sites onto its predicted surface cleft of TSWV N. By N-RNA homology modeling we also discover a new protective feature of N-RNA complexes against the degradation of RNase. Together with the results showing that the N protein forms different orders of oligomers and each oligomer binds to RNA, our findings add new insights into the molecular details of the interaction of TSWV N with RNA components.

The homology model of TSWV N we constructed by I-TASSER showed that the N- and C-terminal domains form a charged surface cleft that has the potential to bind RNA. The 11-nt RNA was modeled and fit well in this electropositive-charged groove. Further alanine substitution analysis confirmed that the positively charged and polar amino acids in the surface cleft of the N protein are indeed responsible for RNA binding. Compared with all other mammalian viruses in the family of Bunyaviridae, *Tospovirus* is the only genus that infects plants (5). Although the amino acid sequence similarity of N protein between TSWV and other mammalian bunyaviruses is relatively low (8–12%), the homology model of TSWV N displays an overall structure similarity with the crystal structures of the N protein of the orthobunyaviruses (Fig. 4, *C* and *D*). Despite the constructed homology model may not be the true structure of N protein, the modeled structures have provided us a basis that we can use for evaluating the structure and analyzing the biological functions of the N protein. Thus, our study demonstrated an excellent example that homology modeling is a useful approach for functional analysis of plant viral proteins.

In the homology model of the N-RNA complexes, the modeled 11-nt RNA component was found to be deeply embedded in the surface cleft of the N protein. Based on this observation, a further RNase A treatment assay revealed that RNA complexed with the TSWV N protein were relatively resistant to the digestion by RNase. Richmond *et al.* (13) have previously reported that the synthetic RNP complexes of TSWV were sensitive to RNase A. In their study they did not indicate the concentration of RNase A used in their study. Their results are still consistent with our data because RNA complexed with the N protein could be digested by the addition of an access amount of RNase (Fig. 7*A*). R94A/R95A or K183A/Y184A mutant that lost the RNA-binding activity was no longer able to protect the RNA from RNase treatment (Fig. 7, *A–C*), suggesting that it is the N protein-RNA interaction that is directly providing resistance to RNase A. The observations that the RNA-N complexes were resistant to RNase treatment have also been reported for the Bunyamwera virus (19, 34, 35). For this mammalian virus, RNA-N complexes were resistant to digestion by reasonable concentrations of RNase A but were digested by >1 μg/ml of RNase A (34, 35). The deep sequestration of RNA within the binding channel was also shown in the structure of the N-RNA complexes from several other mammalian bunyaviruses (19–22). The bound RNA in their N structures is largely inaccessible to RNase. Our findings together with those found in mammalian bunyaviruses lead to the important notion that such N structures that protect RNA from degradation by RNase may be evolutionarily conserved between plant and mammalian bunyaviruses.

In a yeast two-hybrid study, N proteins were shown to interact with each other in a head-to-tail manner (13). In the present study, we demonstrated that recombinant TSWV N protein was able to form monomers, dimers, trimers, tetramers, and pentamers in blue native gel. This feature of the N protein has never been reported. Cross-linking followed by separation on SDS-PAGE further confirmed that the TSWV N protein was able to form a range of higher ordered oligomers. Free-tag N protein also formed the same pattern of oligomeric formation. In native gels, we frequently observe more intensified bands for N trimers. A recent publication by Komoda *et al.* (36) also suggested the presence of N trimers in solution, suggesting trimers may be one of the dominant units for N oligomerization. The N protein of Rift Valley fever virus, a member of the genus *Phlebovirus*, also forms tetrameric, pentameric, or hexameric structures (14, 15). From our homology model of N protein, the oligomerization of TSWV N protein may involve both extensions at the N- and C-arm in organizing adjacent subunits into a higher ordered oligomer assembly. We also found that each type of TSWV N oligomers was able to associate with RNA. Given that the positive-charged amino acids in the surface cleft of the N protein are responsible for RNA binding, oligomerization of the N protein may line up the positive-charged surface cleft from neighboring protomers sequentially, thus forming a continuous RNA-binding channel along the N oligomers. Based on these findings, we propose a model for TSWV RNPs assembly in which a monomer binds to genomic RNAs, followed by binding of another monomer to form a dimer, followed by binding of more monomers to form trimers, tetramers, or higher ordered oligomer to sequentially assemble the RNP complexes.

In conclusion, using homology modeling, we mapped the RNA binding sites onto the predicted surface cleft of the TSWV N protein and gained new insights about the protective function of TSWV N-RNA complexes. The N protein of TSWV has been shown to be a multifunctional protein that is involved in assembly of RNPs (12, 38), viral transcription and replication (5), intracellular (39), and intercellular movement (40, 41), and formation of sphere, enveloped viral particles (37). Our homology models of TSWV N and N-RNA complexes also provide an important basis for further analyzing the biological function of the TSWV N protein in diverse aspects of the TSWV life cycle. Our structure and function analyses of the TSWV nucleocapsid interacting with RNA using homology modeling can also be extended to the study of other plant viruses for which the crystal structure of their viral proteins are not available.

## Footnotes

2 The abbreviations used are:

TSWV: tomato spotted wilt virus
RNP: ribonucleoprotein
Ni-NTA: nickel-nitrilotriacetic acid
DIG: digoxigenin
BCIP/NBP: 5-bromo-4-chloro-3-indolylphosphate/nitro blue tetrazolium
nt: nucleotide
N: nucleocapsid.

## References

[1] Plyusnin A. Beaty B. J. Elliott R. M. Goldbach R. Kormelink R. Lundkvist Å. Schmaljohn C. S. Tesh R. B. (2012) Bunyaviridae. in Virus taxonomy: ninth report of the International Committee on Taxonomy of Viruses (King A. M. Q. Adams M. J. Carstens E. B. Leftkowitc E. J., eds) Elsevier Academic Press, London

[2] Elliott R. M. (1990) Molecular biology of the *Bunyaviridae*. J. Gen. Virol. 71, 501–522 2179464 10.1099/0022-1317-71-3-501

[3] Elliott R. M. (1996) The *Bunyaviridae*. Plenum Press, New York

[4] Goldbach R. D P. (1996) Molecular and biological aspects of *Tospoviruses*. in The Bunyaviridae (Elliott R. M., ed) Plenum Press, New York

[5] Kormelink R. Garcia M. L. Goodin M. Sasaya T. Haenni A. L. (2011) Negative-strand RNA viruses: the plant-infecting counterparts. Virus Res. 162, 184–202 21963660 10.1016/j.virusres.2011.09.028

[6] Adkins S. Quadt R. Choi T. J. Ahlquist P. German T. (1995) An RNA-dependent RNA polymerase activity associated with virions of tomato spotted wilt virus, a plant- and insect-infecting bunyavirus. Virology 207, 308–311 7871744 10.1006/viro.1995.1083

[7] van Knippenberg I. Goldbach R. Kormelink R. (2002) Purified tomato spotted wilt virus particles support both genome replication and transcription *in vitro*. Virology 303, 278–286 12490389 10.1006/viro.2002.1632

[8] Kormelink R. de Haan P. Meurs C. Peters D. Goldbach R. (1993) The nucleotide sequence of the M RNA segment of tomato spotted wilt virus, a bunyavirus with two ambisense RNA segments. J. Gen. Virol. 74, 790 8468562 10.1099/0022-1317-74-4-790

[9] Bucher E. Sijen T. De Haan P. Goldbach R. Prins M. (2003) Negative-strand tospoviruses and tenuiviruses carry a gene for a suppressor of gene silencing at analogous genomic positions. J. Virol. 77, 1329–1336 12502849 10.1128/JVI.77.2.1329-1336.2003 PMC140852

[10] Schnettler E. Hemmes H. Huismann R. Goldbach R. Prins M. Kormelink R. (2010) Diverging affinity of tospovirus RNA silencing suppressor proteins, NSs, for various RNA duplex molecules. J. Virol. 84, 11542–11554 20739523 10.1128/JVI.00595-10 PMC2953175

[11] Kormelink R. (2011) The molecular biology of tospoviruses and resistance strategies. in The Bunyaviridae: Molecular and Cellular Biology (Plyusnin A. Elliott R. M., eds) Horizon Scientific Press, Norwich, UK

[12] Uhrig J. F. Soellick T. R. Minke C. J. Philipp C. Kellmann J. W. Schreier P. H. (1999) Homotypic interaction and multimerization of nucleocapsid protein of tomato spotted wilt *Tospovirus*: identification and characterization of two interacting domains. Proc. Natl. Acad. Sci. U.S.A. 96, 55–60 9874771 10.1073/pnas.96.1.55 PMC15092

[13] Richmond K. E. Chenault K. Sherwood J. L. German T. L. (1998) Characterization of the nucleic acid binding properties of tomato spotted wilt virus nucleocapsid protein. Virology 248, 6–11 9705250 10.1006/viro.1998.9223

[14] Ferron F. Li Z. Danek E. I. Luo D. Wong Y. Coutard B. Lantez V. Charrel R. Canard B. Walz T. Lescar J. (2011) The hexamer structure of Rift Valley fever virus nucleoprotein suggests a mechanism for its assembly into ribonucleoprotein complexes. PLoS Pathog. 7, e1002030 21589902 10.1371/journal.ppat.1002030 PMC3093367

[15] Raymond D. D. Piper M. E. Gerrard S. R. Skiniotis G. Smith J. L. (2012) Phleboviruses encapsidate their genomes by sequestering RNA bases. Proc. Natl. Acad. Sci. U.S.A. 109, 19208–19213 23129612 10.1073/pnas.1213553109 PMC3511139

[16] Raymond D. D. Piper M. E. Gerrard S. R. Smith J. L. (2010) Structure of the Rift Valley fever virus nucleocapsid protein reveals another architecture for RNA encapsidation. Proc. Natl. Acad. Sci. U.S.A. 107, 11769–11774 20547879 10.1073/pnas.1001760107 PMC2900692

[17] Wang Y. Dutta S. Karlberg H. Devignot S. Weber F. Hao Q. Tan Y. J. Mirazimi A. Kotaka M. (2012) Structure of Crimean-Congo hemorrhagic fever virus nucleoprotein: superhelical homo-oligomers and the role of caspase-3 cleavage. J. Virol. 86, 12294–12303 22951837 10.1128/JVI.01627-12 PMC3486442

[18] Guo Y. Wang W. Ji W. Deng M. Sun Y. Zhou H. Yang C. Deng F. Wang H. Hu Z. Lou Z. Rao Z. (2012) Crimean-Congo hemorrhagic fever virus nucleoprotein reveals endonuclease activity in bunyaviruses. Proc. Natl. Acad. Sci. U.S.A. 109, 5046–5051 22421137 10.1073/pnas.1200808109 PMC3324003

[19] Li B. Wang Q. Pan X. Fernández de Castro I. Sun Y. Guo Y. Tao X. Risco C. Sui S. F. Lou Z. (2013) Bunyamwera virus possesses a distinct nucleocapsid protein to facilitate genome encapsidation. Proc. Natl. Acad. Sci. U.S.A. 110, 9048–9053 23569257 10.1073/pnas.1222552110 PMC3670369

[20] Ariza A. Tanner S. J. Walter C. T. Dent K. C. Shepherd D. A. Wu W. Matthews S. V. Hiscox J. A. Green T. J. Luo M. Elliott R. M. Fooks A. R. Ashcroft A. E. Stonehouse N. J. Ranson N. A. Barr J. N. Edwards T. A. (2013) Nucleocapsid protein structures from orthobunyaviruses reveal insight into ribonucleoprotein architecture and RNA polymerization. Nucleic Acids Res. 41, 5912–5926 23595147 10.1093/nar/gkt268 PMC3675483

[21] Niu F. Shaw N. Wang Y. E. Jiao L. Ding W. Li X. Zhu P. Upur H. Ouyang S. Cheng G. Liu Z. J. (2013) Structure of the Leanyer orthobunyavirus nucleoprotein-RNA complex reveals unique architecture for RNA encapsidation. Proc. Natl. Acad. Sci. U.S.A. 110, 9054–9059 23569220 10.1073/pnas.1300035110 PMC3670306

[22] Reguera J. Malet H. Weber F. Cusack S. (2013) Structural basis for encapsidation of genomic RNA by La Crosse Orthobunyavirus nucleoprotein. Proc. Natl. Acad. Sci. U.S.A. 110, 7246–7251 23589854 10.1073/pnas.1302298110 PMC3645531

[23] Arnold K. Bordoli L. Kopp J. Schwede T. (2006) The SWISS-MODEL workspace: a web-based environment for protein structure homology modelling. Bioinformatics 22, 195–201 16301204 10.1093/bioinformatics/bti770

[24] Biasini M. Bienert S. Waterhouse A. Arnold K. Studer G. Schmidt T. Kiefer F. Cassarino T. G. Bertoni M. Bordoli L. Schwede T. (2014) SWISS-MODEL: modelling protein tertiary and quaternary structure using evolutionary information. Nucleic Acids Res. 42, W252–W258 24782522 10.1093/nar/gku340 PMC4086089

[25] Roy A. Kucukural A. Zhang Y. (2010) I-TASSER: a unified platform for automated protein structure and function prediction. Nat. Protoc. 5, 725–738 20360767 10.1038/nprot.2010.5 PMC2849174

[26] Zhang Y. (2008) I-TASSER server for protein 3D structure prediction. BMC Bioinformatics 9, 40 18215316 10.1186/1471-2105-9-40 PMC2245901

[27] Hu Z. Z. Feng Z. K. Zhang Z. J. Liu Y. B. Tao X. R. (2011) Complete genome sequence of a tomato spotted wilt virus isolate from China and comparison to other TSWV isolates of different geographic origin. Arch. Virol. 156, 1905–1908 21805095 10.1007/s00705-011-1078-9

[28] Wu J. Li J. Mao X. Wang W. Cheng Z. Zhou Y. Zhou X. Tao X. (2013) Viroplasm protein P9–1 of Rice black-streaked dwarf virus preferentially binds to single-stranded RNA in its octamer form, and the central interior structure formed by this octamer constitutes the major RNA binding site. J. Virol. 87, 12885–12899 24067964 10.1128/JVI.02264-13 PMC3838130

[29] Yu C. Deng F. Yang C. Wu J. (2008) Prokaryotic expression of full coat protein gene of tomato spotted wilt virus and development of dot-blot ELISA method for this virus detection. J. Zhe Jiang University (Agric. & Life Sci.) 34, 597–601

[30] Niepmann M. Zheng J. (2006) Discontinuous native protein gel electrophoresis. Electrophoresis 27, 3949–3951 16991206 10.1002/elps.200600172

[31] Brindley M. A. Plemper R. K. (2010) Blue native PAGE and biomolecular complementation reveal a tetrameric or higher-order oligomer organization of the physiological measles virus attachment protein H. J. Virol. 84, 12174–12184 20861270 10.1128/JVI.01222-10 PMC2976385

[32] Eubel H. Braun H. P. Millar A. H. (2005) Blue-native PAGE in plants: a tool in analysis of protein-protein interactions. Plant Methods 1, 11 16287510 10.1186/1746-4811-1-11 PMC1308860

[33] Mine A. Takeda A. Taniguchi T. Taniguchi H. Kaido M. Mise K. Okuno T. (2010) Identification and characterization of the 480-kilodalton template-specific RNA-dependent RNA polymerase complex of red clover necrotic mosaic virus. J. Virol. 84, 6070–6081 20375154 10.1128/JVI.00054-10 PMC2876630

[34] Osborne J. C. Elliott R. M. (2000) RNA binding properties of Bunyamwera virus nucleocapsid protein and selective binding to an element in the 5′ terminus of the negative-sense S segment. J. Virol. 74, 9946–9952 11024122 10.1128/jvi.74.21.9946-9952.2000 PMC102032

[35] Mohl B. P. Barr J. N. (2009) Investigating the specificity and stoichiometry of RNA binding by the nucleocapsid protein of Bunyamwera virus. RNA 15, 391–399 19168749 10.1261/rna.1367209 PMC2657012

[36] Komoda K. Narita M. Tanaka I. Yao M. (2013) Expression, purification, crystallization and preliminary x-ray crystallographic study of the nucleocapsid protein of tomato spotted wilt virus. Acta Crystallogr. Sect. F Struct. Biol. Cryst. Commun. 69, 700–703 10.1107/S174430911301302X PMC3668599 23722858

[37] Ribeiro D. Borst J. W. Goldbach R. Kormelink R. (2009) Tomato spotted wilt virus nucleocapsid protein interacts with both viral glycoproteins Gn and Gc in planta. Virology 383, 121–130 18973913 10.1016/j.virol.2008.09.028

[38] Kainz M. Hilson P. Sweeney L. Derose E. German T. L. (2004) Interaction between tomato spotted wilt virus N protein monomers involves nonelectrostatic forces governed by multiple distinct regions in the primary structure. Phytopathology 94, 759–765 18943909 10.1094/PHYTO.2004.94.7.759

[39] Feng Z. Chen X. Bao Y. Dong J. Zhang Z. Tao X. (2013) Nucleocapsid of tomato spotted wilt *Tospovirus* forms mobile particles that traffic on an actin/endoplasmic reticulum network driven by myosin XI-K. New. Phytol. 200, 1212–1224 24032608 10.1111/nph.12447

[40] Kormelink R. Storms M. Van Lent J. Peters D. Goldbach R. (1994) Expression and subcellular location of the NSM protein of tomato spotted wilt virus (TSWV), a putative viral movement protein. Virology 200, 56–65 8128638 10.1006/viro.1994.1162

[41] Soellick T. Uhrig J. F. Bucher G. L. Kellmann J. W. Schreier P. H. (2000) The movement protein NSm of tomato spotted wilt *Tospovirus* (TSWV): RNA binding, interaction with the TSWV N protein, and identification of interacting plant proteins. Proc. Natl. Acad. Sci. U.S.A. 97, 2373–2378 10688879 10.1073/pnas.030548397 PMC15808

